# Assessment of Gauze-Based Negative Pressure Wound Therapy in the Split-Thickness Skin Graft Clinical Pathway—An Observational Study

**Published:** 2011-03-16

**Authors:** Raymond M. Dunn, Ron Ignotz, Trevor Mole, John Cockwill, Jennifer M. Smith

**Affiliations:** ^a^Division of Plastic Surgery, UMASS Medical School, Worcester, Massachusetts, USA; ^b^Smith & Nephew Wound Management, Hull, United Kingdom

## Abstract

**Objectives:** Negative pressure wound therapy (NPWT) is a useful therapy in the preparation of wounds prior to application of a split-thickness skin graft (STSG) both “pregraft” and “postgraft” on top of the STSG. Customarily, a foam-based NPWT has been used, but gauze-based therapy is finding an increasing use. Gauze is easy to apply and forgiving of complicated wound geometries so it can be an ideal material in this indication. The aim of this study was to quantitatively assess the clinical efficacy of gauze-based NPWT as an adjunctive therapy to STSG procedures. **Methods:** A prospective, noncomparative, multicenter evaluation was carried out to assess the performance of gauze-based NPWT. Twenty-one patients had NPWT applied prior to definitive closure by STSG or flap techniques (pregraft group). A further 21 patients underwent an STSG procedure and had gauze-based NPWT placed immediately on top of the STSG (postgraft group). Negative pressure was applied at −80 mm Hg. **Results:** In the pregraft group, NPWT was used for a median of 12 days. Improvement in quality of wound bed with decreased nonviable tissue (from 20% to 0% median wound area) and increased granulation tissue (from 20% to 90% median wound area) was observed. In the postgraft group, median duration of therapy was 5 days at which point median percentage skin graft-take was 96%. **Conclusions:** Gauze-based NPWT appears to be an effective addition to the care and management of wounds intended for definitive closure by STSG.

## INTRODUCTION

Negative pressure wound therapy (NPWT) has become widely used in the treatment of a variety of challenging wounds. One indication where application of NPWT is considered beneficial is in split-thickness skin graft (STSG) procedures where it can provide a number of benefits. Negative pressure wound therapy can be applied to the benefit of graft procedures in 2 distinct stages of the care pathway, described in a series of guidelines presented by the International Panel on Topical Negative Pressure^1^ and outlined later.

First, NPWT can be used to provide rapid wound bed preparation to prepare a wound bed for grafting. In a wound with a significant tissue defect and poor-quality wound bed, NPWT can be used to reduce the size of the defect, increase the quality of the wound bed by increasing the amount of granulation tissue, and contribute toward infection control. Once these goals are achieved, the wound bed may become a good candidate for surgical placement of an STSG. These positive end-points have been observed in numerous studies that describe the closure of a variety of wound types by secondary intention,^2^^-^4 and the ability of NPWT to contribute to wound bed preparation.^5^,^6^

Second, NPWT can be used in place of a bolster dressing following the application of an STSG. Advantages of using NPWT postgrafting include effective removal of serous fluid that can prevent graft take if allowed to accumulate underneath the graft, better immobilization of the graft in anatomically challenging areas, and improved close approximation of the graft to the wound bed, especially where the wound bed has an irregular surface. Several clinical studies have been carried out to evaluate the effect of NPWT when applied on top of an STSG.^7^^-^10 These studies were largely carried out using a polyurethane foam-based NPWT systems either commercially available or improvised kits. Several comparative studies against standard therapy (commonly gauze or bolster dressings) have shown improved clinical outcomes following the use of NPWT applied on top of an STSG, including improved graft take,^7^,^10^ decreased incidence of regrafting procedures,^11^ decreased length of hospitalization,^7^ qualitative appearance of graft,^8^ and improved healing rates^2^,^10^ compared with conventional therapy or standard bolster dressings.

Negative pressure wound therapy consists of a negative pressure source and a wound filler material, which until recently has almost exclusively consisted of a porous polyurethane foam (V.A.C. Granufoam, KCI, San Antonio, Texas). Recently, alternative commercial suppliers have entered the NPWT market and a choice of wound filler materials, including antimicrobial gauze, has become available as a result. In vivo studies have shown that foam and gauze are equally able to transmit negative pressure to the wound bed and equally efficient in promoting changes in microvascular wound blood flow.^12^,^13^ Gauze-based NPWT has been shown to be effective in reducing wound area and volume in a range of difficult wounds.^3^

We hypothesized that gauze-based NPWT could be a useful adjunct to the successful integration of an STSG into the recipient wound bed, in terms of wound bed preparation and in improving STSG take. Gauze is easy to apply and forgiving of complicated wound geometries so it could be an ideal material in this indication. We aimed to assess the effectiveness of gauze-based NPWT system as an adjunctive therapy in skin graft procedures in a prospective, noncomparative clinical evaluation. In particular, we measured the effect on wound area, depth, and volume and calculated the efficiency of take.

## MATERIALS AND METHODS

### Study design

A prospective, noncomparative multicenter evaluation was carried out to assess the performance of gauze-based NPWT in terms of wound progress toward closure wounds deemed suitable for treatment with NPWT (listed on clinicaltrials.gov; ref NCT00994162). The study was performed in accordance with guidelines set forth by the Declaration of Helsinki and approved by the ethical review boards at all involved institutions. All patients provided written informed consent prior to enrollment. Exclusion criteria included the presence of necrotic tissue or more than 25% slough in the wound bed (following removal by debridement these wounds may be eligible), untreated osteomyelitis, malignancy, active bleeding, exposed blood vessels or organs, and untreated wound infection. In total, 153 patients with mixed etiology wounds were assessed. Analysis of the whole population has been published elsewhere.^14^,^15^ A subanalysis on a series of STSG patients was carried out and is described in this report. Wounds healed by STSG were analyzed separately because the clinical outcomes and end-points in this group of patients were different from the remainder of the wounds whose wounds progressed by secondary intention.

### Delivery of gauze-based NPWT

Gauze-based NPWT was delivered using the EZ-Care or V1STA devices (Smith & Nephew, Largo, Florida). These devices were used at a continuous negative pressure of −80 mm Hg. Antimicrobial gauze (Kerlix-AMD; Tyco, Gosport, United Kingdom), provided as part of a tailored dressing kit from the manufacturer, was used to transmit negative pressure to the wound bed using the Chariker-Jeter method of application.^16^ Wounds were examined and dressings changed at 2- to 3-day intervals.

### Clinical procedures

Patients included in this report were divided into 2 groups according to their care pathway, referred to in this report as the pre- and postgrafting groups. Because of the retrospective nature of the data assessment, the 2 groups contained separate patients.

### Pregrafting

Patients with wounds of various etiologies with significant soft tissue deficit and/or poor-quality wound bed were treated with gauze-based NPWT prior to placement of an STSG with the aim of improving the quality of the wound bed and maximizing subsequent STSG success. This group of patients was identified retrospectively from the main patient cohort. All patients who received a graft or a flap following the use of NPWT to prepare the wound were included in the “pregraft” group. Patients who may have been intended for grafting at onset of therapy but who were not grafted for any reason could not be identified and were not included in the analysis. Wounds were assessed, photographs taken, and dressings changed typically every 3 days. The following parameters were measured: wound area, depth, and volume (a function of area and depth); exudate levels (using a 4-point category scoring system—none, mild, moderate, or heavy) and wound bed tissue (measured by recording the percentage area of the wound surface composed of red granulation, yellow slough, yellow fibrous, black necrotic, pink epithelial, and other). Wounds were monitored until they had progressed sufficiently to support the STSG procedure, usually represented by complete coverage of the wound bed with granulation tissue. The end-point in all patients in this group was STSG. In isolated cases, gauze-based NPWT was continued postgrafting. In these cases, only data up to the point at which the graft was placed were assessed.

### Postgrafting

A separate cohort of patients underwent an STSG procedure and had gauze-based NPWT placed immediately on top of the STSG with the aim of stabilizing the STSG immediately postgrafting and improving subsequent graft take. These patients were identified prospectively as a predefined subset of the main clinical evaluation.

A typical procedure was carried out under standard operating room procedures. The wound area was debrided with a scapel to “freshen” the wound bed prior to graft application. The graft was meshed at a 2:1 ratio and stapled to the wound bed. A layer of Acticoat (Smith & Nephew, St Petersburg, Florida) was applied to the graft surface followed by gauze-based NPWT. This dressing remained in place for 4 to 5 days. Upon removal of the NPWT dressing, the graft site was evaluated to ascertain whether the wound was “healed” or “progressing toward healing” (a subjective measure based on clinical judgment) and a separate assessment of the approximate percentage area of successful and unsuccessful graft take was recorded (an objective measure of graft success). Graft sites were subsequently dressed with Xeroform and a gauze dressing. Patients were assessed in the outpatient clinic at 5 to 7 days.

### Data assessment

The 2 groups described earlier were assessed separately in all data analysis. Data relating to patient demographics, comorbidities, wound aetiology, and duration were captured for all patients. Continuous data were summarized using means and standard deviations where the data were normally distributed (eg, patient age) and medians and ranges where the data did not follow a normal distribution (eg, wound duration). Categorical data such as patient sex were summarized using frequency distributions.

## RESULTS

Patient demographics are shown in Table 1. A total of 21 patients were included in the pregrafting group and a further 21 patients were included in the postgrafting group. Both groups included a mixed etiology of wound types including acute and chronic conditions. Results for each group are described separately later.

### Pregrafting

Mean patient age was 51.1 years. Median wound duration prior to placement of gauze-based NPWT was 2.5 weeks (range, 0–104 weeks) (Table 1).

Following application of gauze-based NPWT, therapy was continued until wounds were considered suitable for STSG procedure to proceed as determined by the attending physician. In particular, it was necessary to encourage granulation tissue formation over exposed structures such as exposed tendons. Negative pressure wound therapy was continued for a median of 12 days (range, 5–59 days).

Wound dimensions were monitored over this period (Table 2). Median wound area reduced from 65.6 to 60.5 cm^2^ in this group of patients. Wound depth and volume decreased by a median of 66.7% and 67.3%, respectively, indicating that wounds tended to in-fill from the bottom up with only a small reduction in wound area over the duration of therapy. Median weekly percentage reductions in wound depth and volume of 23.4% and 26.6%, respectively, were calculated.

**Figure 1:**
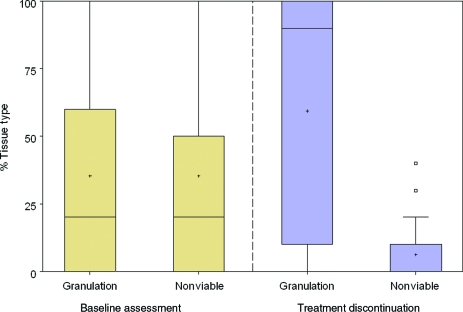
Percentage area of different tissue types in wounds treated with gauze-based negative pressure wound therapy prior to STSG (pregraft group). Percentage wound area consisting of nonviable (necrotic, slough, and fibrous tissue), and granulation tissue, was assessed at baseline (left-hand side) and at treatment discontinuation (right-hand side). Bar within gray box represents median, + represents mean, and □ represents outliers. N = 20.

Analysis of changes in the tissue types present on the wound bed was carried out (Fig 1). Prior to therapy, a median of 20% of the area of the wound bed contained nonviable tissue. Although nonviable tissue was defined as the cumulative value for black necrotic, slough, and fibrotic tissue, in practice because the presence of any black necrotic tissue was a reason for exclusion, at baseline this value represents slough or fibrotic tissue (median percentage area of necrotic tissue at baseline was 0%). Only 20% was composed of healthy granulation tissue. Following gauze-based NPWT, a median value of 90% of the wound surface was composed of granulation tissue, creating a suitable surface on which to place an STSG. A reduction in the amount of nonviable tissue was also observed at treatment discontinuation with a median value of 0% coverage.

When the wound bed was adequately prepared, 15 (75%) of wounds were surgically closed by STSG and 5 (25%) were closed by flap procedures (data not available for one wound).

### Postgrafting

Clinical outcomes following placement of gauze-based NPWT on top of an STSG were assessed in a separate cohort of 21 patients. Mean patient age was 43.2 years. Original wound etiology was mixed (Table 1). Median wound duration prior to graft application was 4.3 weeks (range, 0–26 weeks) and median wound area was 62.8 cm^2^ (range, 4.7–395.8 cm^2^). Following application of gauze-based NPWT on top of the STSG, median time to treatment discontinuation was 5 days at which point 95% of patients were healed or progressing toward healing. One patient (5%) who received a graft to a venous leg ulcer was not classed as “progressing toward healing” because of the presence of fibrotic tissue along with the skin graft. This patient did not, however, need a regraft procedure during the follow-up period.

The degree of graft take was also measured following treatment with gauze-based NPWT and is shown in Table 3. A median graft take of 96% (range, 40–100) was observed. Median duration of stay following the grafting procedure was 5 days (range, 1–15 days). There were no wounds that required regrafting during the study period.

**Figure 2:**
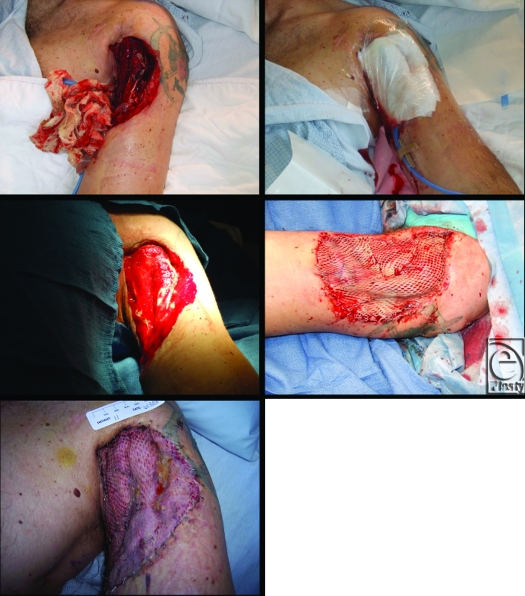
Use of gauze-based negative pressure wound therapy (NPWT) pre- and post-STSG in a trauma patient. Initial application of NPWT was carried out in the operating room and the wound assessment and replacement of gauze-based NPWT occurred at 2 days (A, B). After 8 days of NPWT, the wound was ready for closure with a split-thickness skin graft and NPWT (gauze) was continued for an additional 5 days (C, D). At day 5 postgrafting, NPWT was discontinued with nearly complete graft survival (E).

Figure 2 shows case images from a trauma patient who had suffered a fractured humerus and loss of tissue on upper arm in a motorcycle accident. After 8 days of gauze-based NPWT, the wound was closed with a split-thickness skin graft and NPWT was reapplied for an additional 5 days at which point NPWT was discontinued with nearly complete graft survival. For purposes of data analysis, this patient was included in the “pregraft” group.

## DISCUSSION

Negative pressure wound therapy has been described as a “bridge” technique for wounds that are not amenable to immediate closure by either secondary intention or surgical closure. The majority of publications describe the use of foam-based NPWT. Here the efficacy of gauze-based NPWT in this treatment pathway is described for the first time. Application of gauze-based NPWT to “shallow” graft wounds is hypothesized to be easier to apply because of the highly conformable nature of gauze compared with foam.^17^ Thus, STSG may provide the ideal indication for the adoption of gauze-based NPWT.

One of the principal objectives for use of NPWT in STSG care pathway is the development of a granulating wound bed before grafting to maximize graft take. In the present study, a granulating wound bed was achieved within a median of 12 days of NPWT. This compares well with a previously published study using foam-based NPWT in which diabetic foot ulcer wounds were also successfully prepared for flapping or grafting within 11 days,^18^ significantly faster than in control (standard therapy) wounds. Another advantage of using NPWT before grafting is the rapid weekly reduction of 23% in wound volume. It is possible that the reduction in wound volume induced by NPWT may pave the way from more complex tissue transfers and flap procedures toward simpler, tissue-sparing procedures such as STSG.^5^,^19^ This would have significant impact on patient morbidity.

A high graft take rate of 96% was observed following the use of NPWT on top of an STSG. An identical rate of graft take was reported following 5 days of foam-based NPWT over grafted radial fore-arm flap donor sites.^20^ In the present study, no regraft procedures were required during the study period. A comparative study comparing STSG treated postoperatively with either NPWT or standard bolster dressings, grafts treated with NPWT required significantly fewer repeat grafting procedures than the control (bolster dressing) group.^11^ This implies that gauze-based NPWT may be as efficacious as foam-based NPWT in this indication as well as potentially being easier to apply.^17^

This study describes a retrospective assessment of prospectively captured data. The principal limitation is the lack of a comparator group. Another limitation is that the pregraft and postgraft groups contained separate groups of patients rather than being composed of a single cohort of patients who received continuous use of NPWT both before and after their graft procedure. Negative pressure wound therapy provides a different benefit in the pregraft phase (where improvement of the wound bed and reduction in wound dimensions in particular are important) compared with the postgraft phase (when the bolstering effect and fluid handling capability of NPWT are most important). Because these treatment phases are distinct and well delineated, the use of separate patient cohorts to demonstrate the utility of NPWT in the overall treatment of patients preparing for and receiving STSG is valid although not ideal. Future studies should include a single patient group and follow outcomes during the entire clinical pathway. A further limitation arose from the method of identifying those patients described in the pregraft group. These patients were identified by the fact that they had received an STSG as a defined end-point. All wounds in this group had therefore improved sufficiently to receive an STSG. This study was not able to identify patients where the initial treatment goal may have been to prepare for STSG but where that goal was not achieved. This limitation may have potentially skewed the data in favor of NPWT. Future studies should prospectively identify the treatment goal of applying NPWT and measure outcomes compared with the proportion of patients who achieved their goal. A further limitation is in the subjective nature of some of the wound assessment measurements potentially leading to investigator bias or inter- and intrainvestigator variability.

Currently, application of NPWT over STSG is not considered standard care because of the additional short-term costs associated with its use. However, an evidence base is developing to support the use of NPWT in graft sites at risk of loss or breakdown. Grafts in areas subject to excessive mobility, for example, over a joint^11^,^21^ and highly exuding wounds^21^ are more susceptible to loss. Older patients or those suffering from diabetes mellitus are also known to suffer a higher rate of graft loss^10^,^22^ and may therefore benefit more from application of NPWT.

Application of NPWT to all skin graft patients may also facilitate earlier mobilization that may aid recovery and contribute toward faster hospital discharge. With standard bolster dressings the recommendation is that patients are immobilized for approximately 5 days postoperatively to reduce the risk of graft loss as a result of shearing.^7^,^22^ One limiting factor is the mobility of the NPWT device. Using wall suction as the method of NPWT delivery reduces patient mobility, whereas commercially available devices may significantly improve patient mobility. In the present study, the use of gauze-based NPWT allowed some patients to be discharged much more rapidly than previous STSG procedures would have allowed. Four patients in this study were discharged with a portable pump from hospital, within 24 hours of the graft procedure and managed as outpatients. No difference in outcomes was observed compared to patients who were hospitalized for the full 5 days postoperatively. Further investigation is required to fully appreciate the financial implications of offsetting the costs of therapy against earlier hospital discharge.

The 2 patient groups described in this report represent a holistic care pathway for treatment of wounds intended for definitive closure by STSG improved by the addition of gauze-based NPWT. This study is the first published report describing the effective use of gauze-based NPWT in both the preparation of wounds for STSG and subsequent use as an advanced bolster dressing to enhance graft take.

## CONFLICT OF INTEREST STATEMENT

R.M.D. is a paid consultant of Smith & Nephew. J.M.S., J.C., and T.M. are employees of Smith & Nephew.

## Acknowledgments

We thank the following clinicians for contribution of individual cases that contributed to this study: Paul Savoie, Genevieve Broderick, Julie O'Brien, and Heather Strom (Division of Plastic Surgery, UMASS Medical School, Worcester, Massachusetts), Theresa Hurd (Nursing Practice Solutions, Toronto, Ontario, Canada), Julien Cote (CHUQ Centre Hospitalier Universitaire de Québec), and Chris LaRosa (Virtua Health, New Jersey). We also thank Gary Smith and Alan Rossington for data handling and analysis and Robin Martin for critical review of the manuscript (Smith & Nephew).

This study was funded by Smith & Nephew, who contributed to the study design, data handling, data analysis, and medical writing.

## Figures and Tables

**Table 1:** Patient demographics and wound characteristics*

	Pregraft group	Postgraft group
N	21	21
Male:female	13:8	16:5
Mean age (range); SD	51.1 (31–74); 14.2	43.2 (20–78); 16.2
Principle comorbidities		
Diabetes	23.8%	23.8%
Hypertension	33.3%	14.3%
Original wound etiology		
Chronic	5(23.9%)	4 (16.7%)
Traumatic	9 (42.9%)	7 (33%)
Surgical	7 (33%)	6 (28.5%)
Burn	0	3 (14.3%)
Not recorded	0	1(4.2%)
Median wound duration prior to therapy (weeks)	2.5 (range 0–104)	4.3 (range 0–26)

*Patients were divided into 2 groups according to their positions along the STSG care pathway and treatment goals. The pregraft group received gauze-based negative pressure wound therapy before grafting to improve the wound bed. The postgraft group received gauze-based negative pressure wound therapy immediately after grafting to maximize graft take.

**Table 2:** Median change in dimensions of wounds treated with gauze-based negative pressure wound therapy prior to STSG (pregraft group) (N = 21)

Dimension	At onset of therapy	Immediately prior to grafting	% overall reduction	% Median reduction per week
Wound area, cm^2^	65.6	60.5	0 (Mean = 12.6)	0
Wound depth, cm	1.5	0.5	66.7	23.4
Wound volume, cm^3^	84.8	25.3	67.3	26.6

**Table 3:** Clinical outcomes following application of gauze-based negative pressure wound therapy on top of STSG*

	Median	Range
Graft take (% area)	96	40–100
Length of hospital stay, d	5	1–15

*N = 19 (data not available for 2 patients).
